# Changes in Perceived Stress of Pharmacy Students Pre- and Mid-COVID-19 Pandemic

**DOI:** 10.3390/pharmacy10050114

**Published:** 2022-09-16

**Authors:** Dylan M. Pham, Jia X. Yang, Kelly C. Lee

**Affiliations:** UC San Diego Skaggs School of Pharmacy and Pharmaceutical Sciences, La Jolla, CA 92093, USA

**Keywords:** perceived stress, pharmacy student, COVID-19, work hours

## Abstract

**Objective:** The objective of this study was to examine (1) the difference in perceived stress in first-year pharmacy students before and during the COVID-19 pandemic and (2) the difference in perceived stress among pharmacy students working different numbers of hours. **Methods**: Perceived Stress Scale (PSS), via an electronic survey, was administered throughout 2016–2021 using Qualtrics. End-of-year PSS scores were compared between the pre-pandemic group (2016–2018) and mid-pandemic group (2019–2021) using independent *t*-test and ANCOVA. All analyses were conducted using IBM SPSS Statistic Version 28.0. **Results**: A total of 209 first-year pharmacy students participated (response rate of 88%). No significant difference in mean PSS score was detected in the mid-pandemic cohort when compared to pre-pandemic. The mean PSS score was greater in those who worked greater than 10 h weekly compared to those who worked less. Those who did not work had an even greater mean PSS score than those who worked. **Conclusions:** No significant difference was observed in perceived stress between the pre-pandemic and mid-pandemic cohorts, and an increased perceived stress score was observed in pharmacy students who did not work in comparison to students who worked 1–9 h and 10–29 h.

## 1. Introduction

The long-lasting pandemic of coronavirus disease 2019 (COVID-19) has brought many unprecedented challenges and negative impacts upon students pursuing higher education across the United States [1]. On top of the stressors from their academic programs, students may also experience additional stressors, such as long quarantine duration and infection fears [2]. The high levels of psychological distress from the pandemic have become an international public health concern due to their hazardous effect on mental health [3].

The chronic exposure of the pandemic may lead students to experience psychological discomfort, depression, and anxiety symptoms, and possibly increased risk for suicidal thoughts [4]. Pharmacy students were found to have high level of stress when compared to other health professional students, including medical, dental, and nursing students [1]. Pharmacy students may be even more vulnerable to the detrimental effects of COVID-19 on their mental health.

In March 2020, the World Health Organization declared COVID-19 a global pandemic, which resulted in pharmacy schools transitioning their curriculum from in-person learning to complete virtual learning. We wanted to explore how the pandemic may have contributed to stress among pharmacy students compared to a pre-pandemic period. Furthermore, the pandemic yielded higher demands in pharmacy services, resulting in more students having to work in comparison to pre-pandemic years [5]. We hope to generate knowledge that can aid in the development and implementation of best practices that address the mental well-being of pharmacy students and minimize undesirable health effects of high-stress situations that may occur in the unforeseeable future. We hypothesized that mid-pandemic participants would have a greater end-of-year mean PSS score compared to pre-pandemic participants.

## 2. Methods

The PharmD students at the University of California, San Diego (UCSD) Skaggs School of Pharmacy and Pharmaceutical Sciences (SSPPS) are annually surveyed as part of an ongoing quality assessment of student well-being. Students receive an electronic baseline survey at the beginning of the first year (P1 year) and then receive a survey at the end of each year (P1, P2, P3, P4). The surveys are administered in Qualtrics (Provo, UT, USA). Confidentiality was ensured through student-created 6-digit identifiers. The current study evaluated the end of the year responses for P1 students during academic years 2016–2017 and 2017–2018 to represent the pre-pandemic group and 2019–2020 and 2020–2021 to represent the mid-pandemic group.

The electronic survey included questions about students’ marital status, number of dependents, and hours of paid outside work while in school (ranging in options from working “1–9 h”, “10–29 h”, “did not work”). Students also were asked to rate their general problem-solving skills, time management skills, and study skills using a Likert scale ranging from “poor”, “fair”, “good”, and “very good”. The perceived stress of students was measured using a validated 10-item Perceived Stress Scale (PSS) [6] that measures perceived stress during the last month using a 5-point Likert scale (0 = never, 1 = almost never, 2 = sometimes, 3 = fairly often, 4 = often for questions 1, 2, 3, 6, 9, and 10, and reverse scoring for questions 4, 5, 7, and 8). The mean PSS scores were calculated using the sum of all items and dividing by the number of items [6].

The primary outcome of the study was the comparison of the mean PSS scores between the two study groups (pre-pandemic and mid-pandemic). Secondary outcomes included the comparison of the mean PSS scores among students who worked varying hours and those who self-assessed their general problem solving, time management, and studying skills either poorly (defined as those who responded “poor” or “fair”) or well (defined as those who responded “good” or “very good”).

Descriptive statistics were used (number, %, mean, standard deviation (STD]) to characterize the study groups. The primary outcome was analyzed using the independent *t*-test, using *p*-value < 0.05 as statistically significant. Secondary outcomes were analyzed using ANCOVA with post-hoc comparisons using the Tukey method. All analyses were conducted using IBM SPSS Statistics Version 28.0 (Armonk, NY, USA).

The study was considered exempt by the Human Research Protections Program at UCSD.

## 3. Results

There were 209 P1 students who completed the surveys, with an overall response rate of 88% (79% response rate (102/109) for pre-pandemic, 83% (107/129) for mid-pandemic). No significant differences were found in marital status (*p* = 0.310) and number of dependents (*p* = 0.261) between the pre-pandemic and mid-pandemic cohorts. There were 113 students who reported working in the pharmacy from both pre-pandemic and mid-pandemic cohorts compared to 91 students who did not work. Five students did not report work status.

There were no differences in mean PSS scores between the mid-pandemic group and the pre-pandemic group (Table 1).

Mean PSS scores were also compared among students based upon the number of hours worked weekly (Table 2). Students who did not work had the greatest mean PSS score compared to those who worked 10 to 29 h per week and those who worked 1 to 9 h per week.

**Figure 1:**
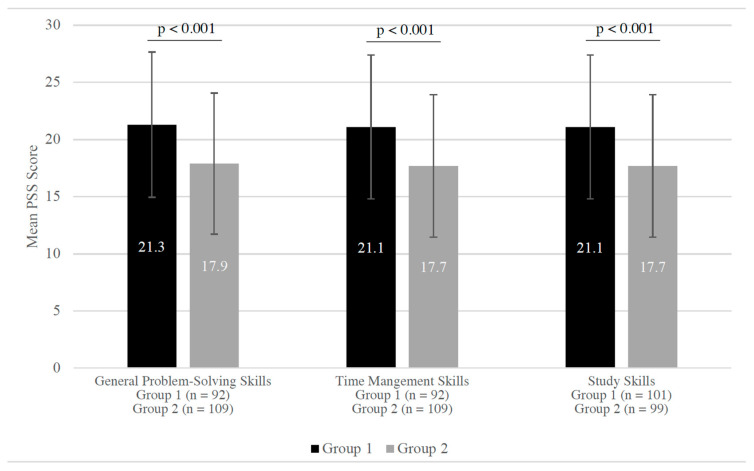
PSS scores between students with different skill sets. Group 1 represents students who rated their general problem-solving skills, time management skills, or study skills as “very poor”, “poor”, or “fair”. Group 2 represents students who rated their general problem-solving skills, time management skills, or study skills as “good” or “very good”. STD = standard deviation.

Students who rated their general problem-solving skills, time management skills, and study skills as “very poor”, “poor”, or “fair” had significantly higher mean PSS scores compared to students who rated their general problem-solving skills, time management skills, and study skills as “good” or “very good” (Figure 1).

## 4. Discussion

We hypothesized that mid-pandemic participants would have a greater end-of-year mean PSS score compared to pre-pandemic participants. There were no differences in mean PSS between the two cohorts in this study; however, the high PSS scores in general were concerning. The mean PSS score of 19.4 from the two cohorts was higher than the national average mean PSS score (11.9–14.2) in a similar age group, as well as the mean PSS score (18.5) in a representative sample of pharmacy students in the US [7,8]. This finding is also similar to the study by Kara et al., who found high levels of perceived stress among health professional students during the pandemic [9]. Hagemeier et al. also found that first-year pharmacy students who transitioned from in-person learning to remote learning during COVID-19 had a significant decrease in well-being scores [10]. The reasons for the higher PSS scores may be due to the increased stress levels of pharmacy students over the last decade due to a lack of jobs [11], postgraduate training opportunities [12], and the financial impact of professional education [13]. There has been a trend within our institution that students are working more hours due to the need for financial stability and potentially favorable application considerations when applying to postgraduate residency positions.

For the secondary aim, we hypothesized that students who worked greater hours weekly would have higher end-of-year mean PSS scores. Our results were somewhat conflicting with our hypothesis in that those who worked 10–29 h did have higher PSS scores than those who worked 1–9 h. There are implicit expectations of students to seek employment during pharmacy school. The increase in demand for pharmacy services during the pandemic may have also negatively impacted students’ stress levels [5,14,15]. A prior study conducted by Sprung et al. showed that work–life imbalance in college students is strongly related to increased perceived stress, anxiety, and depressive symptoms [16]. Another study by Zinurova et al. also showed that higher PSS scores in PGY-1 pharmacy residents were associated with longer working hours [17], which was consistent with our findings. On the other hand, what was surprising in this study was that students who did not work reported a greater mean PSS score compared to both working groups. A possible explanation for this occurrence might be that students who did not work may have had above-average baseline perceived stress scores and were unable to incorporate work into their schedules. Additionally, these students may have been stressed about not meeting institutions’ implicit expectations of seeking employment while attending school [14]. They may also have had higher stress due to financial challenges of not finding employment. Despite these implicit expectations, there still remains a lack of studies investigating stress in pharmacy students in relation to employment. Institutions should conduct further research to implement necessary strategies to manage and cope with the stress of working in conjunction with the rigor of school to promote a healthier work–life balance.

When evaluating students’ general problem-solving skills, time management skills, and study skills, there was a significant increase in mean PSS scores for those who rated themselves as “very poor”, “poor”, or “fair” in these aspects, respectively. The results are consistent with a study by Khatib et al. that found a negative correlation between perceived stress and general time management [18]. A systematic review by Ahmady et al. also suggested that study skills and time management significantly impacted academic achievement, which may explain the significant increase in mean PSS scores in our study [19]. These results suggest that these particular skill sets are critical components to students’ academic functions and potentially their perceived stress levels. Institutions should actively monitor and assist students who are lacking in these skills.

Limitations to this study included not collecting certain demographic variables, including age and gender. Originally, omission of the demographics was intended to preserve confidentiality; however, this information was included in later administrations of the survey so that interventions and specific programs can be tailored to certain demographic groups, if necessary. The study was also limited in sample size; however, the study question evaluating the impact of the pandemic limited the pool of students whose data could be included. The data included in this study were limited to P1 students; PSS scores may differ among students in other years. Additionally, the PSS scale has not been tested during a global pandemic; therefore, the sensitivity to detect changes during such times is unknown.

## 5. Conclusions

The COVID-19 pandemic did not produce significant changes in mean PSS scores in pharmacy students. The end-of-year mean PSS scores in pharmacy students were higher when compared to national samples of individuals in similar age groups. Students who worked longer hours had a significant increase in mean PSS scores. The pandemic is still ongoing with uncertainties regarding long-term consequences, especially in mental health areas. We hope that our findings will aid in implementing evidence-based, innovative strategies and programs that will address the mental health needs not only among current students, but prospective students who are currently enduring the pandemic as well.

## Figures and Tables

**Table 1:** PSS scores between pre-pandemic and mid-pandemic cohorts.

Group	n	Mean PSS Score (STD)	*p*-Value
Pre-Pandemic (2016–2018)	102	19.4 (6.63)	0.472
Mid-Pandemic (2019–2021)	107	19.4 (6.35)	

Range: 0–24 (higher scores indicate higher stress); PSS: perceived stress scale; STD: standard deviation.

**Table 2:** PSS scores based on number of hours worked weekly.

Group	n	Mean PSS Score (STD)	*p*-Value
Group 1 (1–9 h)	74	17.8 (6.37)	0.007
Group 2 (10–29 h)	39	18.8 (5.92)	
Group 3 (did not work)	91	20.9 (6.50)	

Range: 0–24 (higher scores indicate higher stress); PSS: perceived stress scale; STD: standard deviation.

## Data Availability

The data presented in this study are not publicly available due to the confidential nature of the survey responses from current students. Limited data may be available on request from the corresponding author.
